# Reduction of the Ca^2+^ permeability of ligand-gated ion channels as a strategy against excitotoxicity

**DOI:** 10.3389/fncel.2025.1617006

**Published:** 2025-07-16

**Authors:** Tiziano D’Andrea, Sergio Fucile

**Affiliations:** ^1^IRCCS Neuromed, Pozzilli, Italy; ^2^Department of Physiology and Pharmacology, Sapienza Rome University, Rome, Italy

**Keywords:** neuroprotection, neurodegeneration, NMDA receptor, nicotinic acetylcholine receptor, fractional Ca^2+^ current

## Abstract

Excitotoxic damage is due to an excessive Ca^2+^ entry in cells following overactivation of Ca^2+^-permeable ion channels. In neurons, Ca^2+^-dependent excitotoxicity is linked to the prominent activation of N-Methyl-d-Aspartate receptors (NMDARs), exhibiting a high permeability to Ca^2+^. Different neurodegenerative diseases share glutamate-and NMDAR-dependent excitotoxicity as a pathogenic mechanism, but also different ligand-gated ion channels (LGICs) may be involved in excitotoxic-related pathologies, such as muscle nicotinic acetylcholine receptor in some forms of congenital myasthenic syndrome. We posit that excitotoxicity due to the overactivation of Ca^2+^-permeable LGICs may be counteracted by using molecules able to reduce selectively the Ca^2+^ entry, without blocking Na^+^ influx, thus reducing the adverse effects induced by channel blockers. In this review, we recapitulate: (i) the techniques used to quantify the Ca^2+^ permeability of LGICs, with a particular focus on the fractional Ca^2+^ current (P_f_, i.e., the percentage of the total current carried by Ca^2+^); (ii) the known Pf values of the main LGICs; (iii) the modulation of the LGIC P_f_ values induced by drugs and measured to date. These data support the possibility of fighting excitotoxicity-related pathologies with a new therapeutic approach.

## Introduction

Ca^2+^ is the most widespread and versatile intracellular second messenger, used by all cells as a signal to control their activities in response to extrinsic and intrinsic stimuli (Berridge et al., 2000; Bootman and Bultynck, 2020). In particular, the intracellular free Ca^2+^ concentration ([Ca^2+^]_i_) is fundamental in several processes in neuronal physiology: it regulates the release of neurotransmitters from the presynaptic terminals, influencing long-term potentiation (LTP; Grover and Teyler, 1990) and long-term depression (Bolshakov and Siegelbaum, 1994) through N-Methyl-D-Aspartate receptor (NMDAR)-related influx. In neurons, Ca^2+^ regulates gene expression, membrane excitability, dendrite development, synaptogenesis, and many other processes contributing to the neuronal primary functions of information processing and memory storage (Kawamoto et al., 2012). Most of [Ca^2+^]_i_ changes result from influx into the cell regulated through the opening of voltage-dependent Ca^2+^ channels (VDCCs), NMDARs, or other types of ligand-gated ion channels (LGICs) located in the plasma membrane (Kawamoto et al., 2012). Additionally, [Ca^2+^]_i_ levels can be increased by Ca^2+^ release from ER intracellular Ca^2+^ stores via ryanodine receptors (RyRs) and inositol-1,4,5-triphosphate receptors (IP_3_Rs), or by Na^+^-dependent Ca^2+^ efflux (Na^+^/Ca^2+^ exchanger) from mitochondria (Kawamoto et al., 2012). The return to basal [Ca^2+^]_i_ is achieved by Ca^2+^ extrusion or organelle uptake, which uses proteins like sarcoplasmic–endoplasmic reticulum Ca^2+^-ATPase and mitochondrial uniporter (Kawamoto et al., 2012). Moreover, given its importance and potential danger, a high variety of proteins and mechanisms are related to [Ca^2+^]_i_ sensing and buffering. In particular, neurons possess specialized homeostatic mechanisms to ensure a tight command of cytosolic Ca^2+^ levels (Arundine and Tymianski, 2003), such as specialized Ca^2+^-permeable ionic channels, Ca^2+^ pumps, intracellular and intracellular Ca^2+^ buffering systems (Tymianski and Tator, 1996). Any alteration in cellular Ca^2+^ signaling and/or Ca^2+^ homeostasis can lead to serious pathological outcomes and has been linked to the activation of death pathways, which in the Central Nervous System (CNS) cause neuropathologies and neurodegenerative diseases (Sun et al., 2024) sharing excitotoxicity as a fundamental cause, such as amyotrophic lateral sclerosis (ALS; Van Den Bosch et al., 2006), Alzheimer’s disease (AD; Hynd et al., 2004), Parkinson’s disease (PD; Beal, 1998), Huntington’s disease (HD; Sepers and Raymond, 2014), epilepsy (Meldrum, 1993) and schizophrenia (Plitman et al., 2014).

## Excitotoxicity

Ca^2+^-related excitotoxicity is considered one of the key processes of neuropathologies and neurodegenerative diseases (Schlaepfer and Bunge, 1973). Several Ca^2+^-permeable LGICs are involved in excitotoxicity generation, in particular glutamate receptors (Lau and Tymianski, 2010) and ATP P2X receptors (Sáez-Orellana et al., 2016). On the other hand, several studies reported that the activation of neuronal nAChRs (nicotinic acetylcholine receptors) containing the α7 subunit promotes neuronal survival (Dajas-Bailador et al., 2000; Laudenbach et al., 2002). So, despite its fundamental role, an imbalance in glutamate homeostasis and the consequent overactivation of glutamate receptors can damage and kill neurons (Mattson, 2019). Neuronal excitotoxic damage is mostly due to a massive influx of Ca^2+^ through NMDAR (Choi et al., 1988) and VDCCs that cause reactive oxygen species (ROS) release and consequent mitochondrial dysfunction (Mattson, 2003, 2019). Another example of excitotoxic damage happens in the muscular endplate due to rare genetic disorders, such as congenital myasthenic syndromes (CMS; Grassi and Fucile, 2020), which involves mutations in the genes of proteins related to neuromuscular transmission, mostly (about 15%) AChE gene (Beeson et al., 2005; Engel, 2012). Endplate overstimulation causes excitotoxic endplate damage (endplate myopathy) due to the sustained Ca^2+^ influx through *ε*-nAChR-channels, and the Ca^2+^ overloading triggers degenerative events of the endplate structure (Grassi and Fucile, 2014).

Based on the pathogenic concept of overactivation of the excitatory pathways, NMDA receptors have been a longstanding therapeutic target for designing drugs that could be used against these otherwise heterogeneous and complex pathologies (Villmann and Becker, 2007). NMDAR-mediated excitotoxicity seems to start from a signaling mechanism regulated by extrasynaptic NMDARs (Sattler et al., 1999a,b). Conditions that specifically attenuated synaptic NMDAR function did not affect the toxicity caused by exogenous NMDA or glutamate, meaning that extrasynaptic NMDARs must be linked to a molecular pathway triggering neuronal damage (Sattler et al., 1999a,b). Extrasynaptic NMDARs are enriched with the subunit GluN2B, forming GluN2B-containing heterodimers, supporting the hypothesis that extrasynaptic NMDARs constitute a distinct population, serving a specific function; moreover, GluN2B-containing NMDARs have a higher affinity for glutamate, whose concentration is lower in the extrasynaptic space (Papouin and Oliet, 2014). The prevailing theory suggests that synaptic and extrasynaptic NMDAR activation have opposing effects on cell fate: the activity of synaptic NMDARs is thought to favor neuronal survival, via the phosphorylation of intracellular factors such as CREB or Erk1/2; in contrast, cell death is mainly mediated by the activation of extrasynaptic NMDARs, which inhibits the above pathways and promotes the expression of caspase-3, a pro-apoptotic signal (Hardingham et al., 2002; Papouin and Oliet, 2014). To date, the intense research into the mechanisms of excitotoxicity has not enlightened the key intracellular steps responsible for neuronal death in glutamate-dependent excitotoxic processes, mostly because of the heterogeneity of the neurodegeneration that follows glutamate application (Lau and Tymianski, 2010). First, neuronal death in cellular cultures can happen through both apoptosis and necrosis, depending on the severity of NMDA insult: necrotic-like damage predominates in response to acute, relatively intense excitotoxicity (2 mM NMDA), while apoptotic-like neuronal death may develop over many hours after less severe insults, using 300 μM NMDA (Bonfoco et al., 1995). The first to recognize the central role of [Ca^2+^]_i_ in glutamate excitotoxicity-mediated neuronal damage and death was Choi in 1985, demonstrating that excitotoxicity in neuronal cultures was potentiated in a Ca^2+^-rich extracellular solution, while neurodegeneration was markedly reduced in a Ca^2+^-free extracellular solution (Choi, 1985). Furthermore, intracellular Ca^2+^ chelation by cell-permeant molecules such as BAPTA-AM and EGTA-AM protects neurons against excitotoxic injury both *in vitro* and *in vivo* (Tymianski et al., 1994a, 1994b). However, it seems that neuronal mortality is not directly connected to [Ca^2+^]_i_: higher lethality is associated with lower Ca^2+^ influxes via NMDARs compared to higher Ca^2+^ influxes via other Ca^2+^-permeant channels, probably due to the spatial link NMDARs share with neuronal nitric oxide synthase (nNOS), which can produce toxic levels of nitric oxide (NO), through postsynaptic density protein of 95 kDa, the PSD-95 (Chariton and Hafner, 1998; Sattler et al., 1999a,b). Some evidence also supports the idea of free radical generation in mitochondria after the Ca^2+^ influx via NMDARs; radicals, especially superoxide, can interact with other radicals, such as nitric oxide, to form powerful oxidants (Lau and Tymianski, 2010). In fact, the increasing [Ca^2+^]_i_ accompanying NMDAR activation is partially buffered by the intracellular mitochondria, but this Ca^2+^ accumulation causes several dysfunctions, with resultant effects on mitochondrial membrane potential, ATP synthesis and glycolysis (key processes in a cell that consumes a lot of energy, like a neuron), reactive oxygen species generation (ROS) and ultimately failure of cytoplasmic Ca^2+^ homeostasis (Nicholls and Budd, 1998). Moreover, this mitochondrial impairment leads to the release (from the mitochondrial membrane) of Cytochrome C, which causes not only a defect in mitochondrial electron transport (Luetjens et al., 2000) but also caspase-9-dependent caspase-3 activation, which is an “executioner” caspase that leads to neuronal death through the activation of the apoptotic signaling (Seo et al., 2009). More recently, a physical interaction between NMDAR and TRPM4 (a Ca^2+^-impermeant Ca^2+^-activated cationic channel; Rajamanickam et al., 2025) has been shown to be required to observe glutamate-induced excitotoxicity (Yan et al., 2020), introducing a new chapter in the large field of excitotoxicity. All these excitotoxic mechanisms are linked to Ca^2+^ overload, suggesting that avoiding an excessive Ca^2+^ influx could exert neuroprotection.

## Techniques for the quantitative measure of the Ca^2+^ permeability of ion channels

The high relevance of Ca^2+^ influx in physiological processes and its key role in the onset of several neuropathologies and neurodegenerative diseases makes it crucial to understand how to measure and modulate Ca^2+^ entry. The Ca^2+^ permeability of ion channels has been calculated for decades by measuring the channel reversal potential alterations induced by different [Ca^2+^]_o_ (Lewis, 1979), but estimating the Ca^2+^ entry at non-zero electrochemical driving forces, functionally more important, rested on the assumption that there is no interaction among ions passing through the channel (Zhou and Neher, 1993). Then, a new method to quantify the Ca^2+^ flowing through a cationic ion channel was introduced in 1992, involving the simultaneous use of both electrophysiological and Ca^2+^ imaging techniques (Augustine and Neher, 1992): a Ca^2+^ indicator (e.g., Fura-2) is loaded into the cells using a patch micropipette (Zhou and Neher, 1993), and the cell response to an agonist is measured both as a current and a fluorescence emission variation. This mixed approach has been used in the late 90s to measure the P_f_ (the fractional Ca^2+^ current, which represents the percentage of the total current carried by Ca^2+^ ions, at a given membrane potential) of several Ca^2+^ permeable cationic channels: nAChRs (2.5–11.4%, based on species and subunits composition; Zhou and Neher, 1993; Fucile et al., 2003; Fucile, 2004), human AMPAR (from 0.5 to 3.9%, based on subunits composition; (Burnashev et al., 1995) and human NMDAR (from 8.2 to 11%, based on subunits composition). The NMDARs P_f_ is the highest among all the glutamate-gated ion channels currently known (Burnashev et al., 1995). More recently, our group introduced a new way to evaluate the ion influxes through the human NMDARs (and other Ca^2+^-permeable cationic channel) and demonstrate the possibility of modulating the ion selectivity of these receptors, pharmacologically reducing their fractional Ca^2+^ current, causing the consequent reduction of NMDA-mediated Ca^2+^ entry in human neurons (D’Andrea et al., 2024). This approach allows us to record simultaneously the intracellular free Ca^2+^ and Na^+^ concentrations ([Ca^2+^]_i_ and [Na^+^]_i_, respectively D’Andrea et al., 2024). Our innovative approach is no longer centered on directly measuring ionic fluxes, but on the influx-related changes of [Ca^2+^]_i_ and [Na^+^]_i_, measured simultaneously by fluoresce microscopy. This technique, already described in different contexts (Miyazaki et al., 2019), was used by us for the first time to evaluate the LGICs Ca^2+^ permeability.

## Ca^2+^-permeable LGICs and related pathologies

Ca^2+^ ions enter the cells, following their strong concentration gradient, through many kinds of ion channels, exhibiting a large spectrum of functional properties, such as open kinetics, conductance, and ionic selectivity. This wide range of differently regulated pathways, together with the possibility to mobilize Ca^2+^ from intracellular stores, allow cells to strictly regulate the intracellular free Ca^2+^ concentration ([Ca^2+^]_i_), which in turn drives the function of Ca^2+^-dependent proteins and related mechanisms (Berridge et al., 2000). Many Ca^2+^ permeable channels are driven by changes in membrane potential, transducing electrical signals in cellular responses (Zamponi et al., 2015). These VDCCs are highly selective for Ca^2+^ (Tsien et al., 1987), and their activation and inactivation kinetics are crucial to allow Ca^2+^ entry avoiding excitotoxic damage (Christel and Lee, 2012). By contrast, cationic LGICs exhibit a much lower Ca^2+^ selectivity, with Na^+^ being the ion conducting most of the inward current (Burnashev, 1998; Pankratov and Lalo, 2014). Nevertheless, Ca^2+^ flowing through LGICs has a paramount physiological function, in processes such as synaptogenesis (Pagano et al., 2021), synaptic plasticity (Gnegy, 2000), and neuromodulation (Kunz et al., 2013), while excessive activation of these channels has been causally related to relevant pathologies (Sattler and Tymianski, 2001; Spalloni et al., 2013; Guo and Ma, 2021). Obviously, the higher the Ca^2+^ permeability of a channel, the bigger the risk that channel overactivation may lead to excitotoxicity. For these reasons in the last decades many different cationic LGICs have been characterized in terms of their fractional Ca^2+^ current, which is the percentage of the total current carried by Ca^2+^ at a certain membrane potential (Zhou and Neher, 1993; Neher, 1995). These experiments explained how different subunits determine the Ca^2+^ permeability of channels of the same subfamily (Fucile, 2004), and the crucial role of the number and position of electrically charged aminoacidic residues in building the Ca^2+^ selectivity filter (Watanabe et al., 2002; Fucile, 2017). In Table 1 we summarize the known fractional Ca^2+^ currents of different kinds of LGICs, as well as the same parameter measured in mutated channels, some of them resulting in Ca^2+^-related channelopathies. We included in Table 1 only mutated channels with known P_f_ values, but other mutated LGICs with increased Ca^2+^ permeability have been described in pathologies (Lemke et al., 2014; Amin et al., 2018).

**Table 1:** Fractional Ca^2+^ current of cation-selective LGICs.

Extracellular ligand	LGIC subtype	Subunit composition	Species	Functional alteration(s)	Mutation-associated pathology	Pf (%)	Vm (mV)	References
Glutamate	NMDA	GluN1 GluN2A	Rat			11.0	−60	Burnashev et al. (1995)
	NMDA	GluN1 GluN2A	Rat			13.6	−60	Watanabe et al. (2002)
	NMDA	GluN1 GluN2A	Rat			13.5	−60	Jatzke et al. (2002) and Jatzke et al. (2003)
	NMDA	GluN1 GluN2A	Rat			14.1	−60	Egan and Khakh (2004)
	NMDA	GluN1 (D640R) GluN2A	Rat	Reduced P_f_	n.d.	10.6	−60	Watanabe et al. (2002)
	NMDA	GluN1 (D640R) GluN2A	Rat	Reduced P_f_	n.d.	7.0	−60	Jatzke et al. (2003)
	NMDA	GluN1 (R641A) GluN2A	Rat	Increased P_f_	n.d.	14.9	−60	Watanabe et al. (2002)
	NMDA	GluN1 (P642A) GluN2A	Rat		n.d.	12.8	−60	Watanabe et al. (2002)
	NMDA	GluN1 (E643A) GluN2A	Rat	Reduced P_f_	n.d.	10.9	−60	Watanabe et al. (2002)
	NMDA	GluN1 (E644A) GluN2A	Rat	Reduced P_f_	n.d.	11.7	−60	Watanabe et al. (2002)
	NMDA	GluN1 (R645A) GluN2A	Rat		n.d.	13.8	−60	Watanabe et al. (2002)
	NMDA	GluN1 (ARPAAR) GluN2A	Rat	Reduced P_f_	n.d.	7.1	−60	Watanabe et al. (2002)
	NMDA	GluN1 GluN2A	Human			11.3	−70	Plutino et al. (2019)
	NMDA	GluN1 GluN2A	Human			16.2	−60	Moody et al. (2023)
	NMDA	GluN1 GluN2A	Human			9.4	−70	D’Andrea et al. (2024)
	NMDA	GluN1 (D552E) GluN2A	Human	Reduced Q, increased P_f_	Epilepsy	19.4	−60	Moody et al. (2023)
	NMDA	GluN1 (M641I) GluN2A	Human	Increased Q, decreased P_f_	Epilepsy	12.5	−60	Moody et al. (2023)
	NMDA	GluN1 GluN2A (A243V)	Human	Increased Q	Epilepsy	16.6	−60	Moody et al. (2023)
	NMDA	GluN1 GluN2A (N614S)	Human	Increased Q	Epilepsy	15.2	−60	Moody et al. (2023)
	NMDA	GluN1 GluN2A (M705V)	Human	Reduced Q	Epilepsy	14.0	−60	Moody et al. (2023)
	NMDA	GluN1 GluN2A (I814T)	Human	Increased P_f_	Epilepsy	20.9	−60	Moody et al. (2023)
	NMDA	GluN1 GluN2B	Human			9.1	−70	Plutino et al. (2019)
	NMDA	GluN1 GluN2C	Rat			8.2	−60	Burnashev et al. (1995)
	AMPA	GluA1	Rat			3.2	−60	Burnashev et al. (1995)
	AMPA	GluA1(Q)	Rat			3.6	−60	Jatzke et al. (2002) and Jatzke et al. (2003)
	AMPA	GluA1(Q) (R624E)	Rat	Increased P_f_	n.d.	5.3	−60	Jatzke et al. (2003)
	AMPA	GluA1 GluA2(R)	Rat			0.5	−60	Burnashev et al. (1995)
	AMPA	GluA2(Q)	Rat			3.6	−60	Jatzke et al. (2002)
	AMPA	GluA2(Q)	Rat			4.0	−60	Jatzke et al. (2003)
	AMPA	GluA2(Q) (R628E)	Rat	Increased P_f_	n.d.	6.4	−60	Jatzke et al. (2003)
	AMPA	GluA2(N)	Rat			5.3	−60	Jatzke et al. (2002)
	AMPA	GluA2(N)	Rat			5.0	−60	Jatzke et al. (2003)
	AMPA	GluA2(N) (E627R)	Rat	Reduced P_f_	n.d.	1.6	−60	Jatzke et al. (2003)
	AMPA	GluA2(N) (R628E)	Rat	Increased P_f_	n.d.	8.3	−60	Jatzke et al. (2003)
	Kainate	GluA6 (V, C, R)	Rat			<0.2	−60	Burnashev et al. (1995)
	Kainate	GluA6 (V, C, Q)	Rat			1.6	−60	Burnashev et al. (1995)
	Kainate	GluA6(Q)	Rat			2.4	−60	Jatzke et al. (2002)
Acetylcholine	Muscle nicotinic	αβγδ	Mouse			2.1	−50	Ragozzino et al. (1998)
	Muscle nicotinic	αβγδ	Human			2.9	−70	Fucile et al. (2006a)
	Muscle nicotinic	α (V249F)βγδ	Human	Prolonged t_open_	SCMS	3.3	−70	Fucile et al. (2006a)
	Muscle nicotinic	α (G153S)βγδ	Human	Prolonged t_open_	SCMS	2.9	−70	Fucile et al. (2006a)
	Muscle nicotinic	αβεδ	Mouse			4.2	−50	Ragozzino et al. (1998)
	Muscle nicotinic	αβεδ	Human			7.2	−70	Fucile et al. (2006a) and Piccari et al. (2011)
	Muscle nicotinic	αβε*δ*	Human			7.8	−60	Di Castro et al. (2007)
	Muscle nicotinic	α (V249F)βεδ	Human	Prolonged t_open_	SCMS	7.6	−70	Fucile et al. (2006a)
	Muscle nicotinic	α (G153S)βεδ	Human	Prolonged t_open_	SCMS	7.1	−70	Fucile et al. (2006a)
	Muscle nicotinic	αβε (I257F)δ	Human	Prolonged t_open_ decreased P_f_	SCMS	4.6	−70	Piccari et al. (2011)
	Muscle nicotinic	αβε (L269F)δ	Human	Prolonged t_open_ increased P_f_	SCMS	10.2	−70	Piccari et al. (2011)
	Muscle nicotinic	αβε (V259F)δ	Human	Prolonged t_open_ increased P_f_	SCMS	15.4	−60	Di Castro et al. (2007)
	Muscle nicotinic	αβε (T264P)δ	Human	Prolonged t_open_ increased P_f_	SCMS	11.8	−60	Di Castro et al. (2007)
	Neuronal nicotinic	α3β4	Chick			4.4	−50	Lax et al. (2002)
	Neuronal nicotinic	α3β4	Human			2.7	−50	Lax et al. (2002)
	Neuronal nicotinic	α4β2	Chick			2.9	−50	Lax et al. (2002)
	Neuronal nicotinic	α4β2	Chick			3.1	−60	Egan and Khakh (2004)
	Neuronal nicotinic	α4β2	Human			2.6	−50	Lax et al. (2002)
	Neuronal nicotinic	α4β2	Human			3.1	−60	Egan and Khakh (2004)
	Neuronal nicotinic	(β2α4)2 α4	Human			4.0	−70	Sciaccaluga et al. (2015)
	Neuronal nicotinic	(β2α4)2 α5	Mouse			8.8	−70	Sciaccaluga et al. (2015)
	Neuronal nicotinic	(β2α4)2 α5	Human			8.2	−70	Sciaccaluga et al. (2015)
	Neuronal nicotinic	(β2α4)2 α5 (D398N)	Human	Decreased P_open_	Nicotine addiction	8.0	−70	Sciaccaluga et al. (2015)
	Neuronal nicotinic	α7	Rat			8.8	−70	Fucile et al. (2003)
	Neuronal nicotinic	α7	Human			11.4	−70	Fucile et al. (2003)
	Neuronal nicotinic	α7 (L248T)	Human	Increased P_open_, no desensitization	n.d.	6.3	−70	Fucile et al. (2000)
	Neuronal nicotinic	α9α10	Rat			22.0	−70	Fucile et al. (2006b)
Serotonin	5HT	5HT3A	Human			3.5	−70	Martinello et al. (2022)
		5HT3A	Rat			4.7	−60	Egan and Khakh (2004)
	5HT	5HT3A 5HT3B	Human			1.1	−70	Martinello et al. (2022)
ATP	P2X	P2X1	Rat			12.4	−60	Egan and Khakh (2004)
	P2X	P2X1	Human			10.8	−60	Egan and Khakh (2004)
	P2X	P2X2	Rat			5.7	−60	Egan and Khakh (2004)
	P2X	P2X3	Rat			2.7	−60	Egan and Khakh (2004)
	P2X	P2X4	Rat			11.0	−60	Egan and Khakh (2004)
	P2X	P2X4	Human			15.0	−60	Egan and Khakh (2004)
	P2X	P2X5	Rat			4.5	−60	Egan and Khakh (2004)
	P2X	P2X7	Rat			4.6	−60	Egan and Khakh (2004)
	P2X	P2X1 P2X5	Rat			3.3	−60	Egan and Khakh (2004)
	P2X	P2X2 P2X3	Rat			3.5	−60	Egan and Khakh (2004)
	P2X	P2X2 P2X6	Rat			7.7	−60	Egan and Khakh (2004)
	P2X	P2X4 P2X6	Rat			11.3	−60	Egan and Khakh (2004)
pH 5.0, capsaicin		TRPV1	Rat			9.9	−60	Samways et al. (2008)
		TRPV1	Rat			6.6	−60	Samways et al. (2008)

P_f_, fractional Ca^2+^ current; Q, net charge transfer; SCMS, slow channel myasthenic syndrome; P_open_, open probability; t_open_, mean open time; n.d., not described.

## Physiological modulation of the Ca^2+^ permeability of LGICs

The Ca^2+^ permeability of NMDARs can be modulated by physiological processes. Subunits composition of the NMDAR greatly alters several physical and pharmacological properties of the channel: the expression and insertion of the GluN3B subunit, which can co-assemble with endogenous GluN1 and GluN2A and play an important role in modulating synaptic plasticity and neuronal death, reduces the Ca^2+^ permeability of the NMDAR (Matsuda et al., 2003). PKA signaling cascade controls NMDAR Ca^2+^ permeability and NMDAR-mediated [Ca^2+^]_i_ rise in dendritic spines, and in this way PKA directly modulates the induction of NMDAR-dependent LTP at hippocampal Schaffer collateral-CA1 synapses (Skeberdis et al., 2006). NMDAR-mediated Ca^2+^ influx can also be greatly potentiated by the EphB receptor, a member of the Ephrin receptors family, a large family of receptor tyrosine kinases (Takasu et al., 2002).

Recently, GluA2-containing AMPARs, the most abundant in the central nervous system, have been shown to possess a spectrum of different Ca^2+^ permeabilities depending on the subunit composition of AMPAR tetramers, as well as the spatial orientation of transmembrane AMPAR and regulatory proteins and cornichon auxiliary subunits (Miguez-Cabello et al., 2025). The extreme complexity in the physiological Ca^2+^ permeability modulation of Ca^2+^ permeable LGICs demonstrates the importance of those processes and the possibility to alter and modulate the influx of Ca^2+^ through ion channels.

## Pharmacological modulation of the Ca^2+^-permeability of LGICs

Given the high number of Ca^2+^ permeable LGICs, and their causative role in Ca^2+^-related pathologies (calciumopathies; Stutzmann, 2007), we posit the possibility to reduce the noxious impact of their activation by the selective reduction of their Ca^2+^ permeation. We started to investigate this strategy more than a decade ago, analyzing the effect on the P_f_ of the human muscle nicotinic acetylcholine receptor (nAChR) exerted by molecules shown to beneficially act in patients with the slow channel myasthenic syndrome (SCMS; Sadeh et al., 2011). SCMS is a genetic muscle pathology due to mutations of the endplate nAChR dramatically increasing the channel open time and causing a Ca^2+^-dependent degeneration of the endplate (Engel et al., 1982; Sine et al., 1995; Zhu et al., 2015). The same mutations cause a more severe phenotype in human than in rodent animal models, due to the higher Ca^2+^ permeability of the human adult nAChR subtype (containing the e subunit; Gomez et al., 2002; Fucile et al., 2006a). Our data explained why nifedipine, salbutamol and verapamil were able to reduce SCMS symptoms in patients, highlighting their capacity to reduce the fractional Ca^2+^ current through human muscle nAChRs (Piccari et al., 2011). This approach has a crucial aspect: the reduction of Ca^2+^ entry without blocking the channel. This point is extremely relevant, in particular for pathologies due to channel overactivation, in which it is not possible to completely eliminate channel openings. This is true for the endplate, whose proper function requires nAChR opening with precise kinetics: even fast channel mutations produce myasthenia, although without endplate degeneration (Engel et al., 1993). Similar consideration may be done for excessive activation of synaptic and extrasynaptic glutamate-gated ion channels, functionally linked to Ca^2+^-related excitotoxicity and to several forms of neurodegeneration: overactivation is harmful, but block is not possible, leading to severe adverse effects (Hargreaves et al., 1994; Parsons et al., 1999). For this reason, a partial block of NMDAR is one of the few therapeutic options for Alzheimer’s disease, in particular using memantine (partial open channel blocker; Kornhuber et al., 1994; Rammes et al., 2008; Karimi Tari et al., 2024). Despite its neuroprotective potential, memantine is not sufficient to significantly ameliorate AD course (Matsunaga et al., 2015), and other therapeutic approaches must be found. In the last few years, we have focused our attention on the possibility to reduce the P_f_ value of the human NMDAR (Plutino et al., 2019; D’Andrea et al., 2024). We have shown that mild acidosis (pH 6.8–6.5) is not only able to reduce the open probability of NMDARs (as already known; Traynelis and Cull-Candy, 1990), but also to decrease significantly their Ca^2+^ permeability, with a one-third reduction of the P_f_ value (Plutino et al., 2019), further explain the known neuroprotective role of mild acidosis (Tombaugh and Sapolsky, 1990). More recently, we have quantified in terms of P_f_ the observations of Stephen Traynelis group, which investigated the effects of newly developed negative allosteric modulators (NAMs) of NMDARs (Katzman et al., 2015; Hansen et al., 2018). Some of these NAMs were found to reduce the Ca^2+^ permeability of the human NMDAR, measured by the shift of the reversal potential and calculated in terms of PCa/PNa (Perszyk et al., 2021). We measured the P_f_ value of human GluN1/GluN2A NMDARs in the presence of one of these compounds, EU1794-4, showing a significant 40% reduction (D’Andrea et al., 2024). We included in Table 2 all the P_f_ values measured to date, to our knowledge, in the presence of modulators able to reduce the Ca^2+^ permeability of LGICs. This list is rather short.

**Table 2:** Reduction of the fractional Ca^2+^ current of cationic-selective LGICs.

Extracellular ligand	Channel subtype	Subunit composition	Species	P_f_ (%)	Treatment	Reduced P_f_ (%)	Vm (mV)	Therapeutic potential	References
Acetylcholine	Muscle nicotinic	αβεδ	Human	7.2	Nifedipine 10 mM	5.9*	−70		Piccari et al. (2011)
	Muscle nicotinic	αβε (L269F)δ	Human	10.2	Salbutamol 0.1 mM	5.6*	−70	SCMS	Piccari et al. (2011)
	Muscle nicotinic	αβε (L269F)δ	Human	10.2	Salbutamol 10 mM	6.6*	−70	SCMS	Piccari et al. (2011)
	Muscle nicotinic	αβε (L269F)δ	Human	10.2	Propanolol 0.1 mM + salbutamol 0.1 mM	7.3*	−70	SCMS	Piccari et al. (2011)
	Muscle nicotinic	αβε (L269F)δ	Human	10.2	Verapamil 0.5 mM	7.7*	−70	SCMS	Piccari et al. (2011)
	Muscle nicotinic	αβε (L269F)δ	Human	10.2	Verapamil 5 mM	5.4*	−70	SCMS	Piccari et al. (2011)
Glutamate	NMDA	GluN1 GluN2A	Human	11.3	pH 6.8	8.5	−70	Excitotoxicity	Plutino et al. (2019)
	NMDA	GluN1 GluN2A	Human	11.3	pH 6.5	7.6*	−70	Excitotoxicity	Plutino et al. (2019)
	NMDA	GluN1 GluN2A	Human	9.1	pH 6.8	8.1	−70	Excitotoxicity	Plutino et al. (2019)
	NMDA	GluN1 GluN2A	Human	9.1	pH 6.5	4.6*	−70	Excitotoxicity	Plutino et al. (2019)
	NMDA	GluN1 GluN2A	Human	9.4	EU1794-4 30 mM	5.7*	−70	Excitotoxicity	D’Andrea et al. (2024)

P_f_, fractional Ca^2+^ current; SCMS, slow channel myasthenic syndrome. *, statistically significant reduction.

## Discussion

Different therapeutic strategies have been investigated to fight neurodegeneration (see Table 3 for references), but with little impact on clinical applications.

**Table 3:** Investigated therapeutic strategies against neurodegeneration.

Therapeutic strategy	Clinical use	Clinical trial	Effectiveness in animal models	References
NMDAR block	Yes	Yes	Yes	McShane et al. (2019) and Tang et al. (2023)
Antioxidants	No	Yes	Yes	Giordano et al. (2014) and Lee et al. (2020)
ROS scavengers	No	Yes	Yes	Liu et al. (2023) and Houldsworth (2023)
Glutamate scavengers	No	No	Yes	Boyko et al. (2014) and Rogers et al. (2023)
Caspase inhibitors	No	Yes	Limited	Dhani et al. (2021) and Kasana et al. (2024)
Ca^2+^ chelation	No	Yes	Yes	Yeung (2004) and Lees et al. (2013)
Ca^2+^ permeability reduction	No	No	Not tested	D’Andrea et al. (2024)

Our data demonstrate that the selective reduction of Ca^2+^ entry through LGICs is possible, in principle. Reducing Ca^2+^ influx could also lead to adverse effects. Reduced NMDAR-mediated Ca^2+^ signaling can alter physiological Ca^2+^-dependent mechanisms, potentially leading to impaired learning and memory. While excessive NMDAR-mediated Ca^2+^ influx can trigger pathways leading to neuronal death, insufficient Ca^2+^ signaling due to reduced NMDAR activity can also be detrimental: this reduction may contribute to the pathophysiology of neurological diseases, as it can hinder the normal signaling required for neuronal health and function (Painuli et al., 2023). Understanding the processes of NMDAR-mediated Ca^2+^ signaling and its implications for neuronal health will help the design of new drugs, and to maintain a good intracellular Ca^2+^ homeostasis. In this context, different Ca^2+^-targeting approaches may be possible. A chelation-based therapy against stroke, head trauma and neurological damage has been proposed, by using DP-b99, a Ca^2+^/Zn^2+^ chelator derived from BAPTA (Yeung, 2004). Despite a good tolerability in healthy young and elderly volunteers within the dose range evaluated (Rosenberg et al., 2005), DP-b99 showed no evidence of efficacy in treating human ischemic stroke (Lees et al., 2013). In our opinion, a neuroprotective strategy aiming to reduce Ca^2+^ entry through LGIC is worth of experimental attention, being in principle applicable to a wide range of Ca^2+^-related pathologies, especially to the diseases caused by excessive Ca^2+^ entry through LGICs. Many molecules with described neuroprotective effects may indeed be tried for their ability to selectively reduce Ca^2+^ entry, with a particular focus on positively charged compounds already known to reduce the channel (sub)conductance levels. For instance, EU1794-4 reduced the single-channel conductance of NMDAR (Perszyk et al., 2021). With this hypothesis in mind, we measured the P_f_ value of human NMDAR in the presence of memantine, spermine (an intracellular polyamine known to block cation fluxes through LGICs; Tombaugh and Sapolsky, 1990), IEM 1754 and IEM 1460 (open channel blockers; Antonov and Johnson, 1996). None of these molecules was able to reduce the P_f_ of GluN1/GluN2A NMDAR (Plutino et al., 2019), and to date, besides protons, to our knowledge only NAMs developed by the Traynelis groups exhibit this property.

The search for new molecules able to reduce the P_f_ of excitotoxicity-related Ca^2+^-permeable LGICs may produce highly relevant results to improve our pharmacological tools for neuroprotection.
